# Comparison of Effects of Ivabradine versus Carvedilol in Murine Model with the Coxsackievirus B3-Induced Viral Myocarditis

**DOI:** 10.1371/journal.pone.0039394

**Published:** 2012-06-28

**Authors:** Li Yue-Chun, Zhang Teng, Zhou Na-Dan, Ge Li-Sha, Luo Qin, Guan Xue-Qiang, Lin Jia-Feng

**Affiliations:** 1 Department of Cardiology, Second Affiliated Hospital of Wenzhou Medical College, Wenzhou, China; 2 Department of Pediatrics, Second Affiliated Hospital of Wenzhou Medical College, Wenzhou, China; DRFZ, Germany

## Abstract

**Background:**

Elevated heart rate is associated with increased cardiovascular morbidity. The selective I_f_ current inhibitor ivabradine reduces heart rate without affecting cardiac contractility, and has been shown to be cardioprotective in the failing heart. Ivabradine also exerts some of its beneficial effects by decreasing cardiac proinflammatory cytokines and inhibiting peroxidants and collagen accumulation in atherosclerosis or congestive heart failure. However, the effects of ivabradine in the setting of acute viral myocarditis and on the cytokines, oxidative stress and cardiomyocyte apoptosis have not been investigated.

**Methodology/Principal Findings:**

The study was designed to compare the effects of ivabradine and carvedilol in acute viral myocarditis. In a coxsackievirus B3 murine myocarditis model (Balb/c), effects of ivabradine and carvedilol (a nonselective β-adrenoceptor antagonist) on myocardial histopathological changes, cardiac function, plasma noradrenaline, cytokine levels, cardiomyocyte apoptosis, malondialdehyde and superoxide dismutase contents were studied. Both ivabradine and carvedilol similarly and significantly reduced heart rate, attenuated myocardial lesions and improved the impairment of left ventricular function. In addition, ivabradine treatment as well as carvedilol treatment showed significant effects on altered myocardial cytokines with a decrease in the amount of plasma noradrenaline. The increased myocardial MCP-1, IL-6, and TNF-α. in the infected mice was significantly attenuated in the ivabradine treatment group. Only carvedilol had significant anti-oxidative and anti-apoptoic effects in coxsackievirus B3-infected mice.

**Conclusions/Significance:**

These results show that the protective effects of heart rate reduction with ivabradine and carvedilol observed in the acute phase of coxsackievirus B3 murine myocarditis may be due not only to the heart rate reduction itself but also to the downregulation of inflammatory cytokines.

## Introduction

Viral infection of the myocardium produces myocardial necrosis and intense inflammation, which can cause acute heart failure in human and animals [1], [2]. Viral myocarditis is a common cause of acute heart failure, especially in young patients [2]. In patients with heart failure, heart rate (HR) has been shown to be directly related to the risk of cardiac decompensation [3] and overall mortality [4]. An elevated HR may impair ventricular diastolic filling and increase myocardial oxygen demand. The beneficial effects of HR reduction in this setting are well established [5], [6]. β-Blockade is the classical medication to achieve HR reduction, and the main stay of modern therapy for heart failure. Less research has been done for β-blockers in the area of viral myocarditis. We and other investigators have recently reported that carvedilol, a third-generation, nonselective β-adrenoceptor antagonist, reduced myocardial inflammation and necrosis in murine viral myocarditis [7]–[10]. The beneficial action of β-blockade in murine viral myocarditis may be at least partly due to its HR-lowering effect. However, β-blockade also has negative effects (e.g., negative inotropism, and blood pressure reduction), which largely limits their administration in the advent of acute heart failure. The unwanted effects of β-blockers have stimulated the development of drugs which more selectively reduce HR.

Ivabradine, a selective inhibitor of the I(f) channel, reduces resting and exercise HRs without affecting cardiac contractility or blood pressure [11]. Several experimental studies have demonstrated that treatment with ivabradine improves global left ventricular function and reduces cardiac collagen accumulation in rats with congestive heart failure [12]–[14]. In the recent SHIFT trial in patients with symptomatic heart failure and ejection fraction below 35%, ivabradine reduced the primary composite endpoint of cardiovascular death and hospital admission for worsening of heart failure significantly [15]. In the SHIFT echocardiographic substudy [16], ivabradine reverses cardiac remodelling, with significant reductions in left ventricular volumes and an increase in left ventricular ejection fraction (LVEF) in patients with heart failure and left ventricular systolic dysfunction. However, the mechanism for this improvement in left ventricular volumes and function remains to be determined. Recent reports have emphasized that this compound may have pleiotropic effects beyond HR reduction [17], [18]. Not all effects of ivabradine can be reversed by atrial pacing [17], [18]. Ivabradine also exerts some of its beneficial effects by decreasing cardiac proinflammatory cytokines and inhibiting peroxidants and collagen accumulation in atherosclerosis or congestive heart failure [19]–[22]. However, its effects in acute experimental viral myocarditis are unknown. This study therefore was designed to compare the effects of ivabradine and carvedilol in a murine model of acute viral myocarditis induced by coxsackievirus B3 (CVB3).

## Methods

### Murine Viral Myocarditis

Specific pathogen-free inbred, 4-week-old, male Balb/c mice, obtained from Shanghai Laboratory Animal Center, China, were inoculated intraperitoneally with 1.0×10^6^ plaque-forming units (pfu) of CVB3 (strain Nancy) diluted in phosphate-buffered saline to a final volume of 0.1 ml. Group control was inoculated intraperitoneally 0.1 ml with normal saline solution. The day of virus inoculation was defined as day 0. All experiments were carried out in accordance with China Animal Welfare Legislation and were approved by the Wenzhou Medical College Committee on Ethics in the Care and Use of Laboratory Animals.

### Drug Administration

Ivabradine and carvedilol were obtained from Servier Co. (Courbevoie, France) and Roche China Co. (Shanghai, China), respectively. Starting 24 h after infection ivabradine (10 mg/kg per day, n = 40) and carvedilol (10 mg/kg per day, n = 40) were administered by gavage for 14 consecutive days, whereas control group (n = 30) and myocarditis group (n = 40) mice received the normal saline solution in the same way. Eight surviving mice from each group were killed on day 4, 7 or 14.

### Hemodynamic Measurements

HR and blood pressure (BP) were measured using a photoelectric tail cuff detection system (softron BP-98A from Japan) on conscious mice that had been pre-warmed for 10 minutes at 37°C in a thermostatically controlled heating cabinet. The values were averaged from at least three consecutive readings on each occasion.

### Echocardiographic Examination

Transthoracic echocardiography was performed using a Sonos 5500 ultrasound machine (Phillips, USA.) with a 12 MHz phased array transducer, real time digital acquisition, storage, and review capabilities, as previously described [8]. The transducer was covered with a surgical latex glove finger filled by ultrasound transmission gel to provide a standoff of 0.5–0.7 cm. The transducer was used at a depth setting of 2 cm to optimize resolution. Mice were anaesthetized with intraperitoneally with 3% chloral hydrate (0.01 ml/g). The chest was shaved. Mice were placed on a heating pad in a shallow left lateral position. Two-dimensional, M-mode, Doppler flow images were obtained in parasternal long-axis view. The left ventricular end-systolic and end-diastolic internal diameters (LVESd, LVEDd) were measured over the course of at least 3 consecutive cardiac cycles. The LVEF and fractional shortening (FS) were then both calculated.

### Plasma Noradrenaline

Plasma noradrenaline was measured using high-performance liquid chromatography and electrochemical detection. After mice were anesthetized intraperitoneally with pentobarbital (50 mg/kg), the arterial blood samples were taken and centrifuged at 3000 g for 15 min. The plasma were stored at −80°C for subsequent determination of noradrenaline concentration.

### Survival Rate

Survival was measured over a 14-day period.

### Myocardial Histopathology

The ratio of heart weight to body weight (HW/BW) was calculated. The heart tissue was fixed in 10% formalin, embedded in paraffin, sectioned, and stained with hematoxylin and eosin. Several sections of each heart were scored blindly by two observers. The scores assigned to these specific sections were averaged. The extent of cellular infiltration and myocardial necrosis was graded and scored as follows: 0 =  no lesion; 1+  =  lesions involving <25% of the myocardium; 2+  =  lesions involving 25% to 50%; 3+  =  lesions involving 50 to 75%; and 4+  =  lesions involving 75% to 100%.

### RNA Isolation and Reverse Transcriptase-polymerase Chain Reaction (RT-PCR)

Total RNA, frozen in liquid nitrogen, was extracted from the myocardial samples by the Trizol method (Invitrogen, Carlsbad, CA, USA) according to the manufacturer’s instructions. cDNA was synthesized by reverse transcription using total RNA (3 µg) as a template. Semiquantitative RT-PCR was used to detect the mRNA abundance of monocyte chemoattractant protein-1 (MCP-1), interleukin (IL)-6, tumor necrosis factor (TNF)-α, intercellular adhesion molecule-1 (ICAM-1) and vascular cell adhesion molecule-1 (VCAM-1) (Table 1). Additionally, RT-PCR was also used to detect the CVB3 RNA abundance in the infected myocardium. The mRNA abundance was quantified as optical densities (OD) equalized with β-actin mRNA levels using a BandScan 5.0 software (Glyko, Novato, CA, USA). Nucleicacid sequences of all PCR products were confirmed to be identical to published GenBank data.

**Table 1:** Primer sequences used for semi-quantitative RT-PCR.

mRNA	Primers	Annealing temperature (°C)	Cycles	Product (bp)
CVB3	F-CGGTACCTTTGTGCGCCTGTR-CAGGCCGCCAACGCAGCC	61	30	314
MCP-1	F-GCCAACTCTCACTGAAGCCR-GCTGGTGAATGAGTAGCAGC	50	30	161
IL-6	F-TGCTGGTGACAACCACGGCCR-GTACTCCAGAAGACCAGAGG	60	33	308
TNF-α	F-CCTGTAGCCCACGTCGTAGCR-TTGACCTCAGCGCTGAGTTG	50	31	374
ICAM-1	F-CAACTGGAAGCTGTTTGAGCTGR-TAGCTGGAAGATCGAAAGTCCG	59	33	437
VCAM-1	F-CCTCACTTGCAGCACTACGGGCTR-TTTTCCAATATCCTCAATGACGGG	60	34	442
β-actin	F-AGGGAAATCGTGCGTGACATR-CATCTGCTGGAAGGTGGACA	55	24	450

### Assay of Cytokine Levels in the Heart

Cytokine levels were measured with various enzyme-linked immunosorbent assay (ELISA) kits manufactured by Westang Biotech Co Ltd (Shanghai,China) for MCP-1, IL-6 and TNF-α. The sensitivity of the kit is 8 pg/ml for MCP-1, 16 pg/ml for IL-6, and 13 pg/ml for TNF-α. Cytokine levels are expressed as pg/mg of heart.

### Detection of Malondialdehyde (MDA) and Superoxide Dismutase (SOD) Contents in Cardiac Homogenates

The myocardium was homogenized in 9 (v/v) volumes of ice-cold PBS, and the homogenates were centrifuged at 3000×g for 15 min at 4°C to obtain the supernate. MDA and SOD in cardiac homogenates were measured by assay kits, respectively, according to the manufacturer’s instructions. The kits were manufactured by Jiancheng Co. (Nanjing, China).

### Detection of Apoptosis

Apoptosis was detected in myocardial tissue sections using the terminal transferase-mediated DNA nick end labelling (TUNEL) assay. Apoptotic cells were identified using an *in situ* cell death detection kit, pod (Roche, Switzerland). The myocardial tissue sections were incubated with 50 µl TUNEL reaction mixture containing terminal deoxynucleotidyl transferase (TdT) for 60 minutes at 37°C. After incubating with horseradish peroxidase (HRP)-avidin (0.5 mg/ml in phosphate buffered saline) at 37°C for 30 minutes, they were stained with DAB and observed in 10 randomly selected fields by microscope. Nuclei with brown staining indicated TUNEL positive cells. Cell death was expressed as a percentage of total cells counted.

### Statistical Analysis

All values were expressed as mean value ± standard error (SE). Survival rate was analyzed by the Kaplan-Meier method. Statistical analysis was performed by one way analysis of variance (ANOVA), followed by Fisher protected least significant difference test. A value of P<0.05 was considered significant.

## Results

### Hemodynamics of Mice

**Figure 1:**
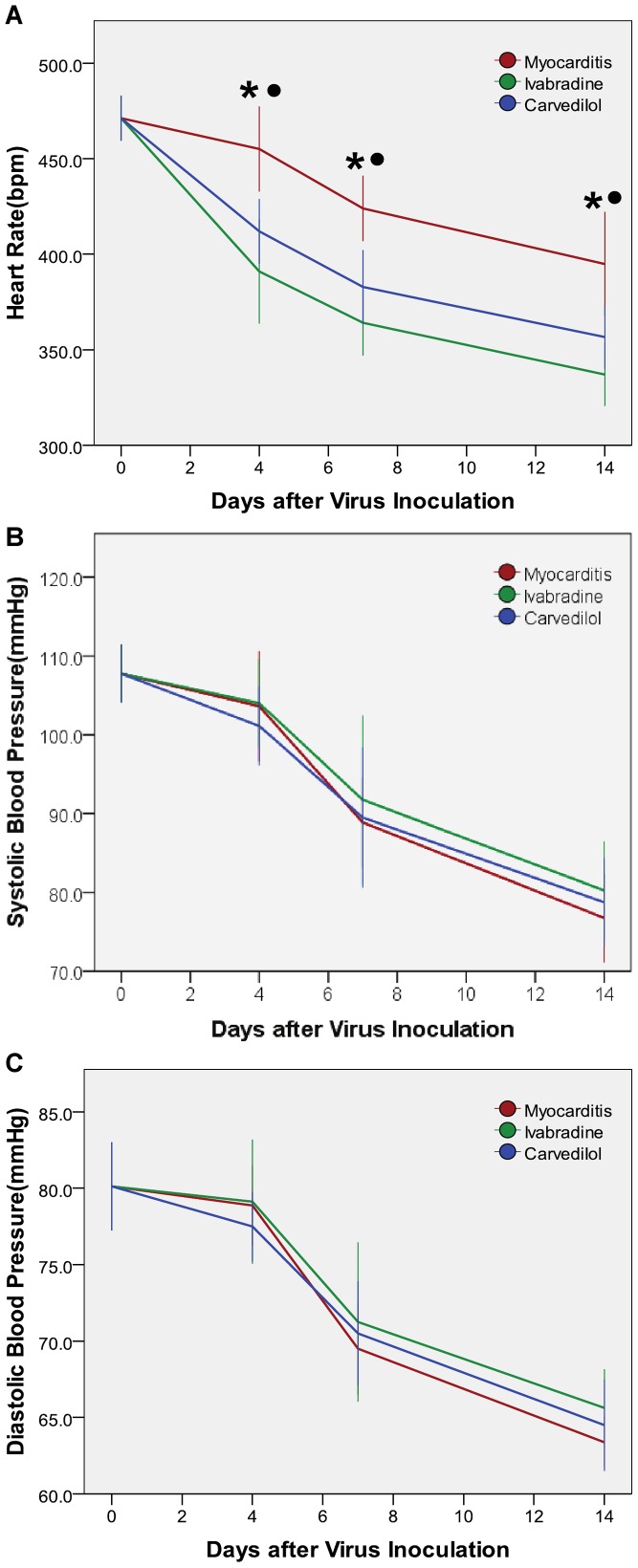
Heart rate and blood pressure measured on days 4, 7 and 14. *P<0.05 ivabradine versus myocarditis; ^•^P<0.05 carvedilol versus myocarditis.

HRs in the ivabradine and carvedilol groups were significantly decreased compared with the normal and myocarditis groups on days 4, 7 and 14 (Figure 1A), but there was no significant difference between the ivabradine and carvedilol group. Systolic blood pressure (SBP) in the ivabradine group and carvedilol group and myocarditis group were significantly decreased compared with the normal group on days 4, 7 and 14 (Figure 1B). Systolic blood pressure (SBP) of the carvedilol group and the ivabradine group slightly increased compared to the myocarditis group on days 7 and 14, but this effect did not reach statistical significance, and SBP of the ivabradine group did not differ compared to the carvedilol group. No differences in diastolic blood pressure (DBP) were found among the ivabradine, carvedilol and myocarditis groups on days 4, 7 and 14 (Figure 1C).

### Echocardiographic Findings

**Figure 2:**
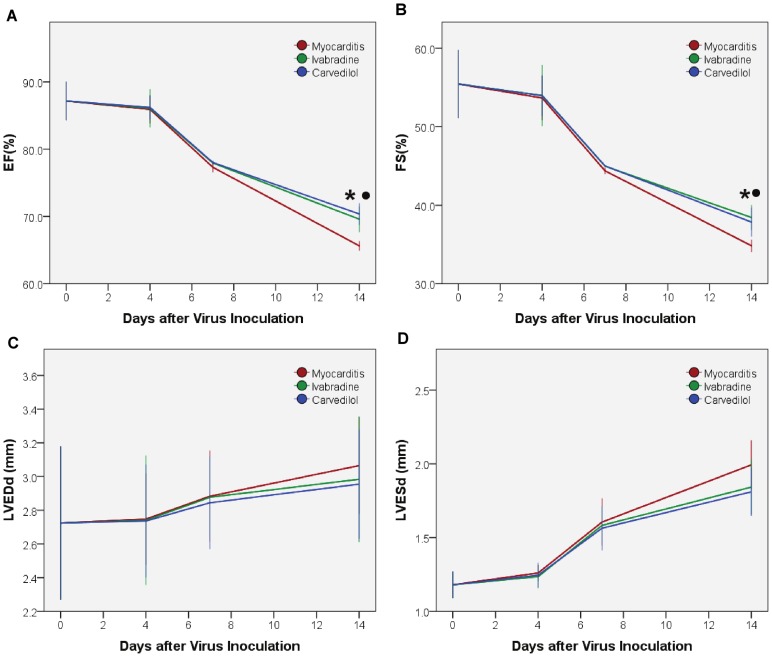
Echocardiographic study on days 4, 7 and 14. EF, left ventricular ejection fraction; FS: fractional shortening; LVEDd, left ventricular end-diastolic diameter; LVESd, left ventricular end-systolic diameter. *P<0.05 ivabradine versus myocarditis; ^•^P<0.05 carvedilol versus myocarditis.

EF and FS in the ivabradine, carvedilol and myocarditis groups were significantly decreased compared with the normal group on days 4, 7 and 14. No differences in EF and FS were found among the ivabradine, carvedilol and myocarditis groups on days 4 and 7 (Figure 2A–B). EF and FS of the ivabradine and carvedilol groups significantly increased compared to the myocarditis group on day 14, but those of the ivabradine group did not differ compared to the carvedilol group. LVESd in the ivabradine, carvedilol and myocarditis groups were significantly increased compared with the normal group. No differences in LVEDd and LVESd were found among the ivabradine, carvedilol and myocarditis groups on days 4, 7 and 14 (Figure 2C–D).

### Survival Rate

**Figure 3:**
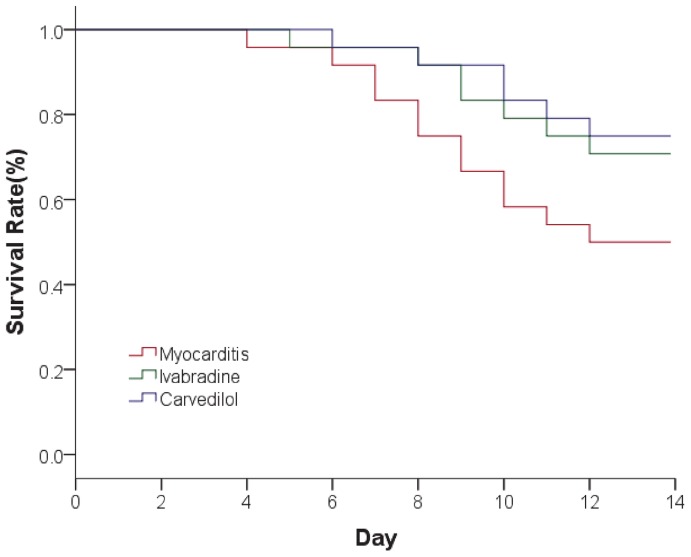
The survival rate in CVB3-inoculated mice followed for 14 days. Both ivabradine and carvedilol slightly increased the survival rate in CVB3 infected mice.

The survival rate in CVB3-inoculated mice followed for 14 days was 50.0% for those treated with saline, 70.8% for those treated with ivabradine, and 75.0% for those treated with carvedilol (Figure 3). The survival rate increased in the ivabradine group and carvedilol group compared to the myocarditis group, but this effect did not reach statistical significance.

### Plasma Noradrenaline Levels

**Figure 4.The:**
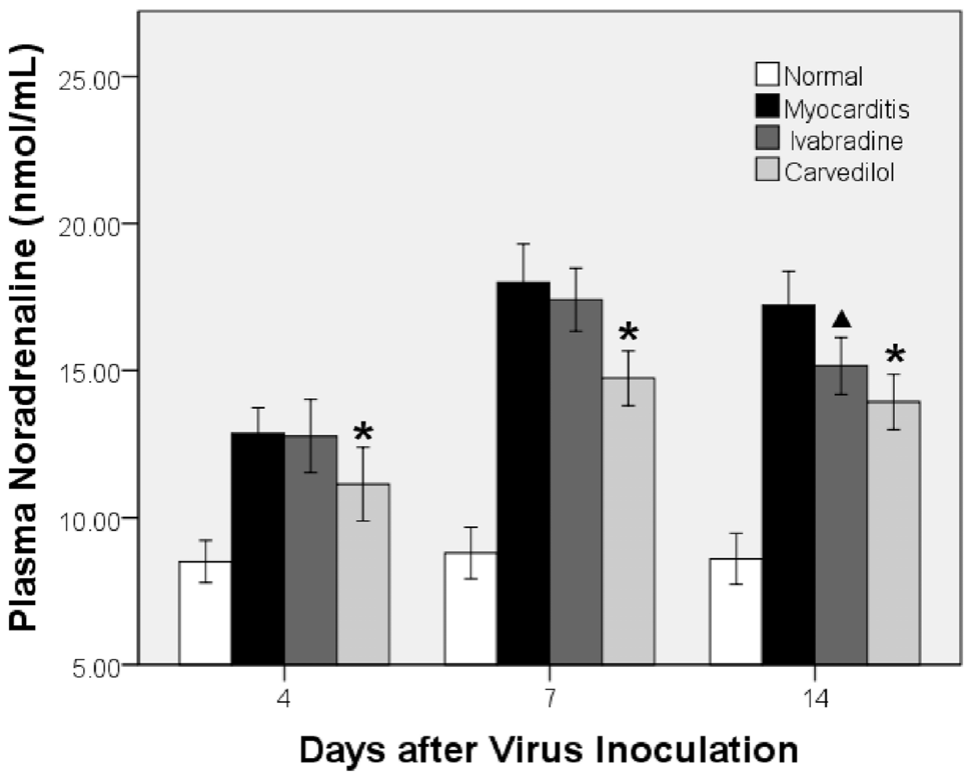
effects of ivabradine and carvedilol on plasma noradrenaline on days 4, 7 and 14. *P<0.05 versus myocarditis; ^▴^P<0.05 ivabradine versus myocarditis.

Plasma noradrenaline levels were significantly higher in the ivabradine, carvedilol and myocarditis groups than in the normal group on days 4, 7 and 14 (Figure 4). Noradrenaline of the carvedilol group significantly decreased compared to the myocarditis group on days 4 and 7, but that of the ivabradine group did not differ compared to the myocarditis group. Plasma noradrenaline of the ivabradine and carvedilol groups significantly decreased compared to the myocarditis group on days 14, and no difference in plasma noradrenaline were found between the ivabradine and carvedilol groups on day 14 (Figure 4).

### HW/BW Ratio

Four, 7 and 14 days after infection, the myocarditis group showed a reduction in BW and HW and an increase in HW/BW ratio. The HW/BW ratio was significantly lower in the ivabradine and carvedilol groups than in the myocarditis groups on days 7 and 14. No differences in HW/BW ratio were found between the ivabradine group and carvedilol group (Table 2).

**Table 2:** Effects of ivabradine and carvedilol on HW/BW on days 4, 7 and 14.

Group	n	HW/BW (10^−3^)		
		4d	7d	14d
Normal	8	4.35±0.18	4.47±0.09	4.76±0.15
Myocarditis	8	4.82±0.12*	5.84±0.10*	6.59±0.21*
Ivabradine	8	4.77±0.16*	5.25±0.08* ^,^ †	5.55±0.112* ^,^ †
Carvedilol	8	4.72±0.10	5.07±0.09* ^,^ †	5.43±0.07* ^,^ †

* P<0.05 versus normal.

† P<0.05 versus myocarditis.

### Myocardial Histopathology

**Figure 5:**
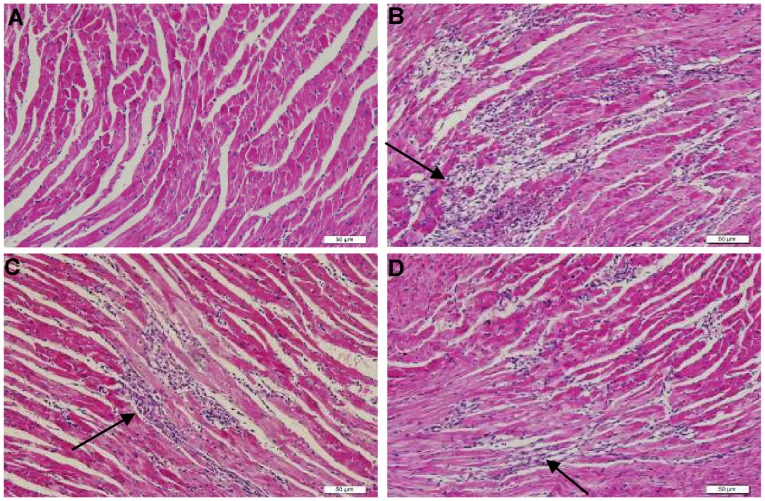
Histopathology in the heart (Hematoxylin Eosin×200). (A) Histopathology in a normal group (grade 0). (B) Representative histopathology in a myocarditis group. Several large foci of cellular infiltrations (arrow) in the inflammatory region are shown (grade 3). (C) Representative histopathology in a ivabradine group. Several small foci of cellular infiltrations in the inflammatory region (arrow) are shown (grade 2). (D) Representative histopathology in a carvedilol group. Several small foci of cellular infiltrations in the inflammatory region (arrow) are shown (grade 2).

On days 4, 7 and 14 at sacrifice, severe injuries to myocardium with cellular infiltration in the myocarditis group were observed. The severity of cellular infiltration were significantly reduced in the ivabradine group and carvedilol group compared with the myocarditis group on days 7 and 14 (Figure 5, Table 3), indicating a significantly reduced severity of disease. No differences in the pathologic scores were found between the ivabradine group and carvedilol group.

**Table 3:** Effects of ivabradine and carvedilol on myocardial histopathology on days 4, 7 and 14.

Group	n	Infiltration			Necrosis		
		4d	7d	14d	4d	7d	14d
Normal	8	ND	ND	ND	ND	ND	ND
Myocarditis	8	1.38±0.18	2.63±0.26	1.88±0.13	1.00±0.19	1.88±0.13	2.38±0.18
Ivabradine	8	1.25±0.16	1.87±0.23*	1.13±0.13*	0.88±0.13	1.3±0.18*	1.63±0.18*
Carvedilol	8	1.13±0.13	1.63±0.26*	1.0±0.00*	0.75±0.16	1.1±0.13*	1.25±0.16*

ND, not detected.

* P<0.05 versus myocarditis.

### Gene Expression of Cytokines in the Heart

**Figure 6:**
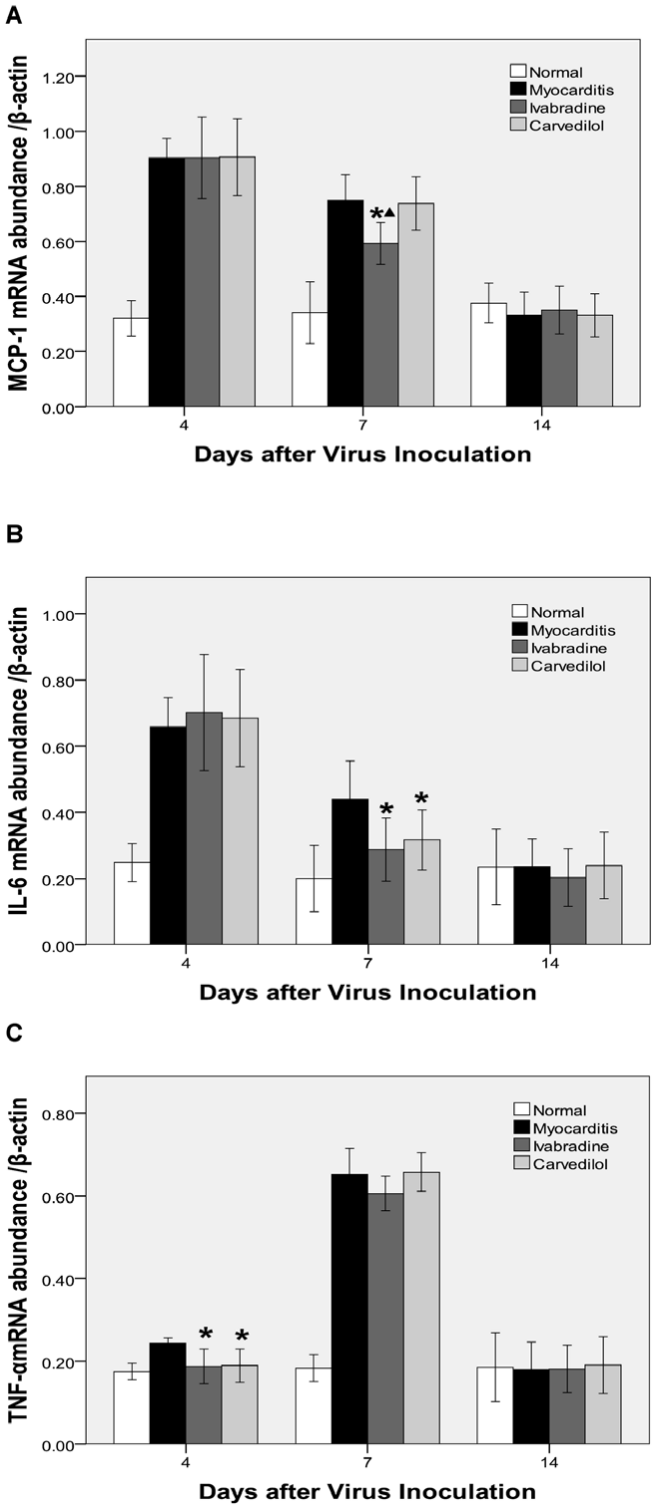
Expression of cytokine mRNAs (A: MCP-1; B: IL-6; C: TNF-α) in the myocardial tissues of mice on days 4, 7 and 14. *P<0.05 versus myocarditis; ^▴^P<0.05 versus carvedilol.

**Figure 7:**
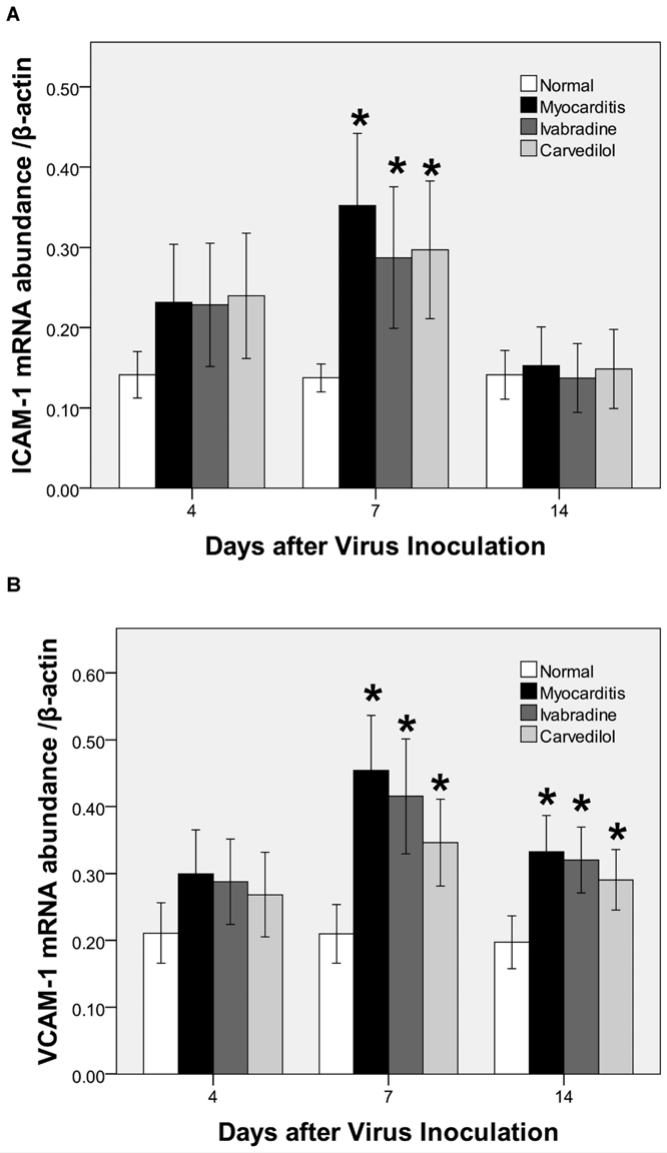
Expression of cytokine mRNAs (A: ICAM-1; B: VCAM-1) in the myocardial tissues of mice on days 4, 7 and 14. *P<0.05 versus normal.

On days 4 and 7, the mRNA levels of the MCP-1, IL-6, TNF-α, ICAM-1 and VCAM-1 in the myocardium of the infected mice were significantly upregulated compared with the normal group. On day 4, cardiac TNF-α. levels were significantly downregulated in the ivabradine group and carvedilol group compared with the myocarditis group (Figure 6). On day 7, the mRNA levels of the IL-6 were significantly lesser in the ivabradine group and carvedilol group compared with the myocarditis group (P<0.05; Figure 6), and ivabradine administration attenuated the increase in MCP-1 significantly (P<0.05) in the infected mice, but carvedilol had no effect on the MCP-1. Ivabradine and carvedilol treatment both led to no significant reduction in ICAM-1 and VCAM-1 compared to the untreated infected mice on day 7 (Figure 7). On day 14, no differences in the mRNA levels of the MCP-1, IL-6, TNF-α., ICAM-1 and VCAM-1 were found among the ivabradine group and carvedilol group and myocarditis group.

### ELISA Analysis of Cytokines Levels in the Heart

**Figure 8:**
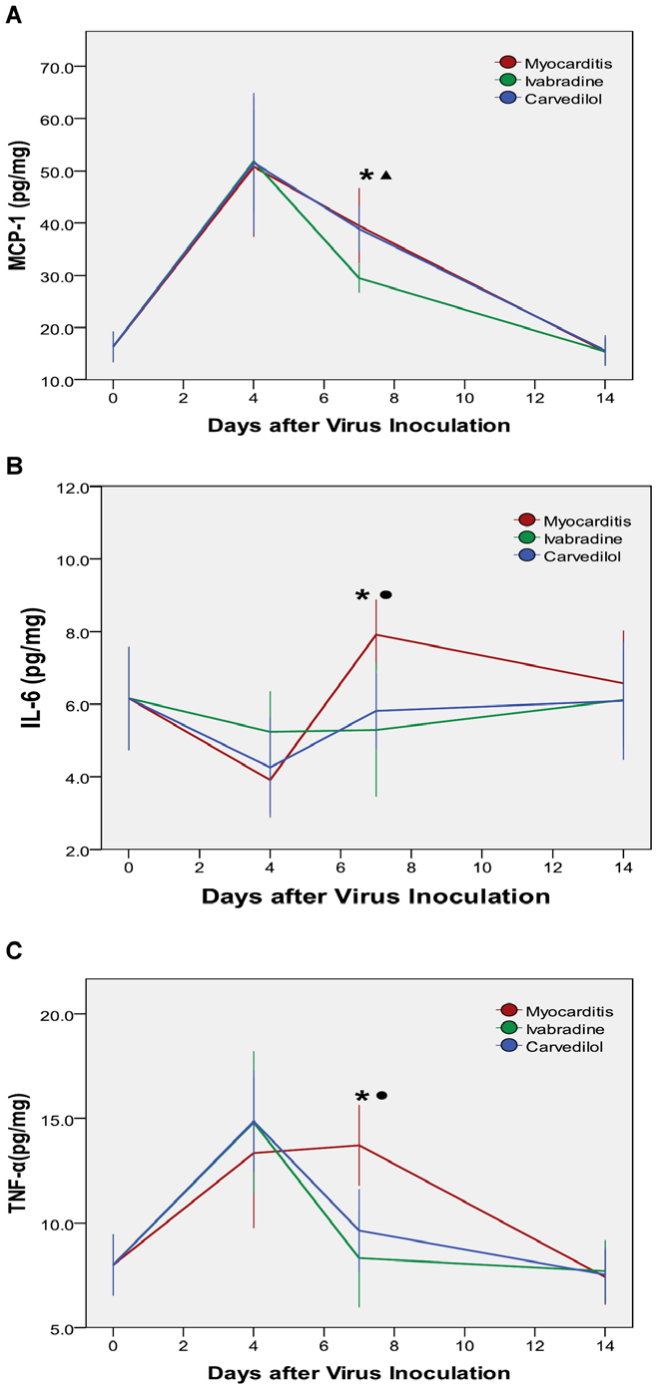
Cytokine levels measured by ELISA analysis (A: MCP-1; B: IL-6; C: TNF-α) in the myocardial tissues of mice on days 4, 7 and 14. *P<0.05 ivabradine versus myocarditis; ^•^P<0.05 carvedilol versus myocarditis;^ ▴^P<0.05 ivabradine versus carvedilol.

On day 4, there were no significant differences in the cytokine levels among the ivabradine group and carvedilol group and myocarditis group. On day 7, the levels of TNF-α and IL-6 were significantly lower in the ivabradine group and carvedilol group compared with the myocarditis group (P<0.05; Figure 8), and the levels of MCP-1 were significantly reduced by ivabradine (P<0.05; Figure 8), but carvedilol had no effect on the MCP-1. On day 14, no differences in the levels of the MCP-1, IL-6 and TNF-α. were found among the ivabradine group and carvedilol group and myocarditis group.

### Viral Genome in the Myocardium

**Figure 9:**
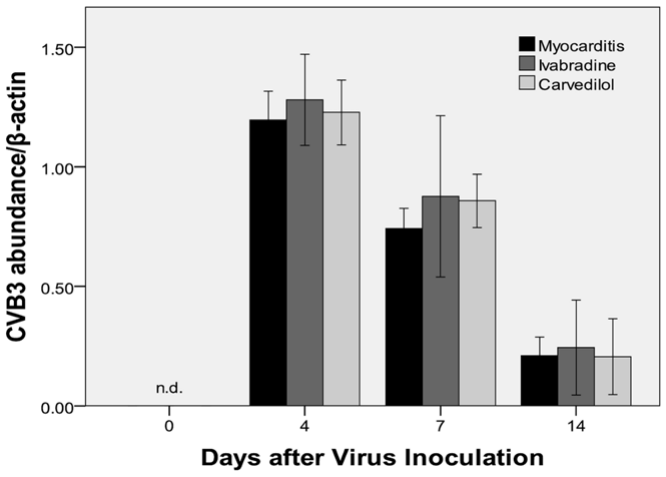
CVB-3 RNA abundance in the infected myocardium of mice on days 4, 7 and 14. nd, not detected.

CVB3-RNA abundance by semiquantitative RT-PCR-analysis were found in the myocardium of the infected mice on days 4, 7 and14. Ivabradine treatment and carvedilol treatment both produced a slightly higher CVB3-RNA abundance in the infected myocardium compared to the myocarditis group on days 4 (1.28±0.09, 1.23±0.07 vs 1.19±0.06; Figure 9) and 7 (0.88±0.16, 0.86±0.06 vs 0.74±0.04; Figure 9). There were no significant differences in the CVB3-RNA abundance among the ivabradine group and carvedilol group and myocarditis group on day 14.

### MDA Contents in the Myocardium

**Figure 10:**
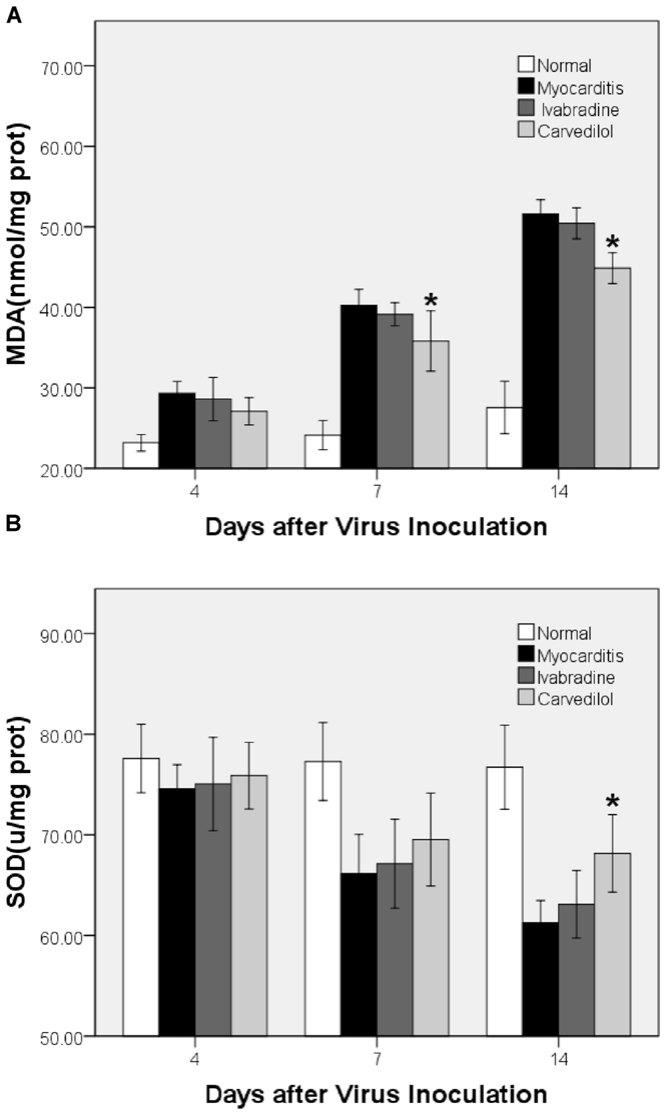
The effects of ivabradine and carvedilol on MDA and SOD on days 4, 7 and 14. *P<0.05 carvedilol versus myocarditis.

The myocardial MDA contents in the myocarditis group were significantly higher than those in normal control group (Figure 10). On days 4, 7 and 14, there were no differences in the myocardial MDA contents between the myocarditis group and the ivabradine group. On days 7 and 14, carvedilol manifestedly decreased the contents of MDA.

### SOD Contents in the Myocardium

On days 4, 7 and 14, no differences in the myocardial SOD contents were found between the ivabradine and myocarditis groups (Figure 10). On day 14, the contents of SOD were significantly higher in the carvedilol group than in the myocarditis groups.

### Cardiomyocyte Apoptosis

**Figure 11:**
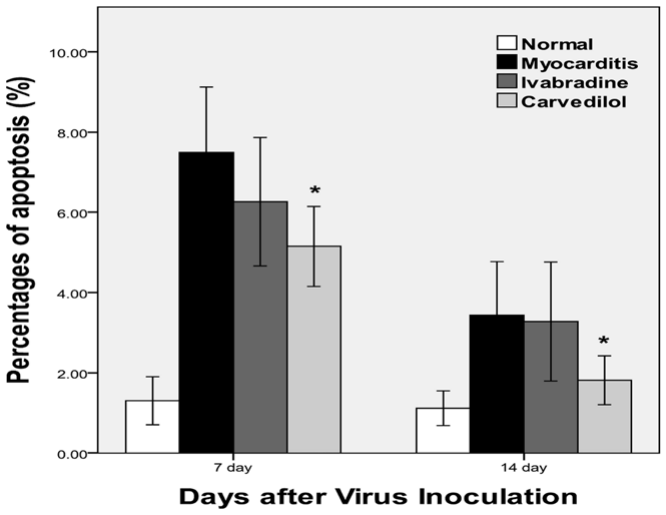
The percentages of apoptosis in the uninfected and infected myocardium of mice on days 7 and 14. *P<0.05 carvedilol versus myocarditis.

**Figure 12:**
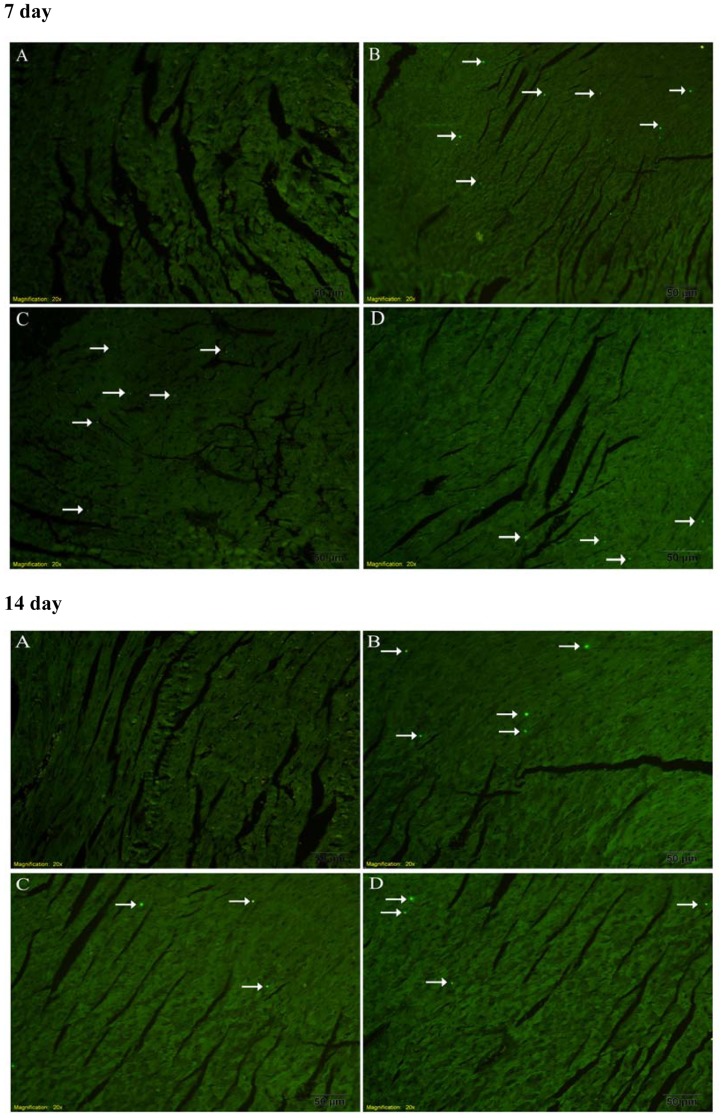
Detection of apoptotic cardiomyocytes with the TUNEL assay. Apoptosis cell was indicated by a small white arrow. (A) normal group. (B) myocarditis group. (C) ivabradine group. (D) carvedilol group.

The TUNEL positive cells in the myocardium of the infected mice were significantly increased compared with the normal mice. Carvedilol treatment attenuated the increase in percentages of apoptosis significantly (P<0.05) in the infected mice, whereas ivabradine only slightly reduced the percentages of apoptosis in the infected mice compared with the myocarditis group (Figure 11). The TUNEL positive cell nuclei appeared condensed and rounded, showing typical features of apoptotic morphology (Figure 12).

## Discussion

The novel finding of the study is the marked amelioration of acute viral myocarditis after selective HR reduction with the I(f) channel inhibitor ivabradine in a CVB3 murine myocarditis model. Ivabradine and carvedilol treatment result in almost identical effects. To the best of our knowledge, this is the first study to investigate the effects of ivabradine in acute viral myocarditis. In this study, ivabradine, a pure HR-lowering agent, and carvedilol, a β-blocker, similarly effectively reduced HR, attenuated myocardial lesions and improved the impairment of left ventricular function. In addition, ivabradine treatment as well as carvedilol treatment showed significant effects on altered myocardial cytokines, with a decrease in the amount of plasma noradrenaline. The increased myocardial MCP-1, IL-6, and TNF-α. in the infected mice were significantly attenuated in the ivabradine treatment group. Ivabradine had no significant anti-oxidative and anti-apoptotic effects in CVB3-infected mice. These results show that the protective effects of HR reduction with ivabradine and carvedilol observed in the acute phase of CVB3 murine myocarditis may be due not only to the HR reduction itself but also to the downregulation of inflammatory cytokines. The findings also show ivabradine to be non-inferior to carvedilol in the setting of myocarditis.

### Effects of Ivabradine on Myocardial Cytokines and Oxidative Stress

Several reports have suggested that ivabradine may have an beneficial effect on cardiovascular inflammation [19], [21]. Recently, Custodis et al. demonstrated that ivabradine decreased inflammatory cytokines in apolipoprotein E deficient mice [19]. Schirmer et al. reported that ivabradine modulated inflammatory cytokine gene expression and increased endothelial nitricoxide synthase (eNOS) activity in a murine model of hypercholesterolemic atherosclerosis [21]. In support of these findings, treatment with ivabradine in CVB3-infected mice caused a significant improvement in left ventricular function, combined with marked changes in cytokines expression especially MCP-1, IL-6 and TNF-α. suppression. It has been suggested that cytokines exert an important role in the pathophysiology of viral myocarditis [23]–[27]. MCP-1 is critically linked to various inflammatory diseases [28]. MCP-1 stimulates an inflammatory response in cardiomyocytes by enhancing IL-6 levels and is involved in the pathogenesis of myocarditis [25], [29]. IL-6 and TNF-α are both involved in the pathogenesis of myocarditis and may induce advanced cardiac dysfunction [24], [26], [27]. In this study, ivabradine attenuated myocardial inflammatory lesions and downregulated the levels of MCP-1, IL-6 and TNF-α. The results suggest that ivabradine exerts its therapeutic effects in part by suppressing the production of MCP-1, IL-6 and TNF-α. The decrease in inflammatory cytokines by administration of ivabradine may be caused by several mechanisms. First, the effect of ivabradine on myocardial inflammation is mainly attributed to the reduction in HR. Increased HR is known to be associated with systemic inflammation [30]. A reduction in HR will prolong diastolic filling time, and thus may improve ventricular filling and myocardial O_2_ supply, and decrease O_2_ consumption in impaired left ventricular function [12]. By preventing myocardial hypoxia, ivabradine may diminish the production of cytokines such as IL-6 and TNF-α in congestive heart failure [12], [31]. Second, the improvement in the left ventricular filling by the reduction in HR may inhibit the sympathetic activity. In this study, the plasma noradrenaline levels in the ivabradine-treatment group were lower than the untreated infected group on day 14. These data are in agreement with a earlier study demonstrating that ivabradine significantly reduced noradrenaline levels in rats with left ventricular dysfunction [12]. In recent years, it has become clear that catecholamines influence the production of cytokines [32]–[35]. Therefore, ivabradine may limit the effect of noradrenaline to prevent the catecholamine-induced production of cytokines in acute viral myocarditis.

Previous observations have identified anti-oxidative effects as the underlying mechanisms of the beneficial effect of ivabradine in atherosclerosis [19], [36]. Selective HR reduction with ivabradine was associated with decreases in NADPH oxidase activity, superoxide production and lipid peroxidation in the vascular wall in a previous study conducted in ApoE−/− mice fed with a cholesterol-rich diet for 6 weeks [19]. Drouin et al. found that ivabradine limited cardiac dysfunction and prevented the renovascular and cerebrovascular endothelial dysfunction in dyslipidaemic mice, which was associated with the antioxidant effects of ivabradine [22]. Kröller-Schön et al. also showed that ivabradine improved vascular endothelial function, which was associated with decreased vascular reactive oxygen species production due to reduced NADPH oxidase activity and the prevention of eNOS uncoupling [36]. However, several studies found that ivabradine had no beneficial effect on oxidative stress [20], [21], [36]. Kröller-Schön et al. reported that ivabradine had no effect on endothelial function and vascular reactive oxygen species production in angiotensin II-treated rats [36]. In a hypercholesterolemic rabbit model, ivabradine did not attenuate circulating levels of lipid peroxidation [20]. Similarly, concentrations of reactive oxygen species were unchanged with ivabradine treatment in a murine model of hypercholesterolemic atherosclerosis [21]. In the present study, ivabradine had no significant anti-oxidative effects on the CVB3-infected myocardium. The discrepancy between the ivabradine effects on oxidative stress might relate to the different pathophysiological mechanisms in these different models, the determined tissue (vessel, circulating plasm, or myocardium), the treatment duration, or animal species.

### Comparison of the Effects of Ivabradine versus Carvedilol

In the present study, both ivabradine and carvedilol similarly and significantly reduced HR, increased LVEF and improved left ventricular systolic function. However, mechanisms behind this protection were different. Recently, we and other investigators demonstrated that carvedilol, but not metoprolol (a selective β_1_-adrenoceptor antagonist), reduced the severity of acute viral myocarditis in mice, which might be due to its immunoregulatory and antioxidant effects [7]–[10]. The antioxidant activity of carvedilol has been attributed to the carbazole moiety of the drug [37]. In agreement with the previous studies, we found that carvedilol exerted some of its beneficial effects by downregulating the production of IL-6 and TNF-α in the present study. Moreover, carvedilol, but not ivabradine, had significant anti-oxidative effects in CVB3-infected mice in the present study. The beneficial action of carvedilol in murine viral myocarditis may be mainly due to its blocked β-adrenergic-stimulating effects of catecholamines and antioxidant properties, and partly due to its HR-lowering effect [7]–[10]. Although ivabradine probably mimics in part the effect of β-blockers in the study, the key mechanism by which ivabradine exerts these effects is the reduction in HR per se. The reduction in HR with ivabradine may prolong diastolic filling time, improve the O2 supply/demand ratio and thus improve ventricular filling and stroke volume. Moreover, ivabradine lowers HR without any negative inotropic or lusitropic effect, thus preserving ventricular contractility. Therefore, the decrease in inflammatory lesions and the improvement in cardiac function with ivabradine treatment is probably due not only to the HR reduction itself but also to the downregulation of inflammatory cytokines. The CVB3 RNA abundance was performed using semiquantitative RT-PCR. The CVB3 RNA abundance in the ivabradine and carvedilol groups was both slightly higher than the untreated infected mice in the present study. The degree of myocardial inflammation seems to be more important than CVB3 RNA abundance for the depression of left ventricular dysfunction in the murine model with the CVB3-induced viral myocarditis [38]. In addition, it has been suggested that cardiomyocyte apoptosis may be involved in the course of experimental viral myocarditis [39], [40]. In agreement with previous studies [39], [40], we found that the percentages of apoptosis in the myocardium of the infected mice were higher than in the uninfected mice. The interesting finding was that treatment with carvedilol significantly reduced the apoptosis in the myocardium of the infected mice, and ivabradine had no significant anti-apoptotic effects. This indicates that a pure HR reduction does not account for the effects of β-blockade in experimental viral myocarditis.

Cardiac vascularization is of importance in the model of myocarditis. The cardiac vascularization can be stimulated by long-term HR reduction [41], [42]. Several previous studies have shown that ivabradine have beneficial effects on the capillarization of heart in experimental models of post-myocardial infarction heart failure and hypercholesterolemic atherosclerosis [21], [43]. Therefore, the improvement of cardiac vasculature may represent a therapeutic advantage in myocarditis as well. Additional studies to determine the effects of ivabradine on the cardiac capillarization in experimental viral myocarditis are needed.

### Ivabradine and Large Clinical Trials

A number of experimental studies have demonstrated that ivabradine have an beneficial effect on left ventricular remodeling in the failing heart [12]–[14]. In recent large clinical trials, ivabradine improved survival in patients with ischemic and nonischemic causes of heart failure [15], [16]. SHIFT trial was designed to evaluate the effect of HR reduction with ivabradine on cardiovascular outcomes, symptoms, and quality of life in patients with symptomatic heart failure and LVEF≤35% including ischemic and nonischemic causes. The primary composite endpoint of cardiovascular death or hospital admission for worsening heart failure was significantly reduced, largely due to a highly significant reduction in hospitalization from heart failure. The study confirmed HR as an important target in heart failure, and showed that selective reduction of HR with ivabradine can improve outcomes [16]. In our study, ivabradine and carvedilol similarly effectively attenuated myocardial lesions and improved the impairment of left ventricular function caused by viral myocarditis.The results of our experiment may not be directly relevant to the outcomes of clinical trials. To date there is no clinical trial that is conducted with ivabradine therapy for patients with viral myocarditis. Therefore, further experimental and clinical studies would be welcome in future.

In conclusion, these findings indicate that both ivabradine and carvedilol have a therapeutic benefit in murine CVB3-induced myocarditis. The cardioprotection of ivabradine and carvedilol may be due not only to the HR reduction itself but also to the downregulation of inflammatory cytokines.
